# Effects of Selective Dry Cow Treatment on Intramammary Infection Risk after Calving, Cure Risk during the Dry Period, and Antibiotic Use at Drying-Off: A Systematic Review and Meta-Analysis of Current Literature (2000–2021)

**DOI:** 10.3390/ani11123403

**Published:** 2021-11-29

**Authors:** Jim Weber, Stefan Borchardt, Julia Seidel, Ruben Schreiter, Frederike Wehrle, Karsten Donat, Markus Freick

**Affiliations:** 1 Veterinary Practice Zettlitz, 09306 Zettlitz, Germany; markus.freick@htw-dresden.de; 2 Clinic for Animal Reproduction, Faculty of Veterinary Medicine, Free University of Berlin, 14163 Berlin, Germany; Stefan.Borchardt@fu-berlin.de; 3 Faculty of Agriculture/Environment/Chemistry, HTW Dresden—University of Applied Sciences, 01326 Dresden, Germany; jseidel67@yahoo.de; 4 ZAFT e.V., Center for Applied Research and Technology, 01069 Dresden, Germany; ruben.schreiter@htw-dresden.de; 5 Animal Health Service, Thuringian Animal Diseases Fund, 07745 Jena, Germany; fwehrle@thtsk.de (F.W.); kdonat@thtsk.de (K.D.); 6 Clinic for Obstetrics, Gynecology and Andrology of Large and Small Animals with Veterinary Ambulance, Justus Liebig University Giessen, 35392 Giessen, Germany

**Keywords:** bacteriological culture, cattle, mastitis, udder health

## Abstract

**Simple Summary:**

The vast majority of all antibiotics used in the dairy industry are applied to control mastitis, one of the most important infectious diseases in dairy cows. The aim of this systematic review and meta-analysis is to evaluate the efficacy of selective dry cow treatment vs. blanket dry cow treatment (i.e., antibiotic treatment of all quarters in all cows) on different measures of udder health as well as antibiotic use at drying-off. In this way, it could contribute to minimizing antimicrobial agent usage, which is increasingly criticized based on a growing spread of antimicrobial resistance and its negative consequences for public health.

**Abstract:**

The objectives of this paper were (i) to perform a systematic review of the literature over the last 21 yr and (ii) to evaluate the efficacy of selective dry cow treatment (SDCT) vs. blanket dry cow treatment (BDCT) in dairy cows regarding the risk of intramammary infection (IMI) after calving, new IMI risk after calving, cure risk during the dry period, and a reduction in antibiotic use at drying-off by meta-analysis. The systematic search was carried out using the databases PubMed, CAB Direct, and ScienceDirect. A meta-analytical assessment was performed for each outcome of interest using random-effects models, and the relative risk (RR) for IMI and cure or the pooled proportion for antibiotic use was calculated. The final number of included studies was *n* = 3 for IMI risk after calving and *n* = 5 for new IMI risk after calving, cure risk during the dry period, and antibiotic use. The RR levels for IMI (RR, 95% confidence interval [CI]: 1.02, 0.94–1.11; *p* = 0.592), new IMI (RR, 95% CI: 1.06, 0.94–1.20; *p* = 0.994), and cure (RR, 95% CI: 1.00, 0.97–1.02; *p* = 0.661) did not differ significantly between SDCT and BDCT. Substantial heterogeneity was observed between the trials regarding the pooled proportion of antibiotic use within the SDCT groups (*I*^2^ = 97.7%; *p* < 0.001). This meta-analysis provides evidence that SDCT seems to be an adequate alternative to BDCT regarding udder health with a simultaneous reduction in antibiotic use. Limitations might arise because of the small number of studies included.

## 1. Introduction

Mastitis is one of the most important infectious diseases in dairy cows worldwide, responsible for substantial economic losses and detrimental effects on ruminant welfare arising from reduced milk yield and fertility as well as increased use of antimicrobial agents and higher culling risk [1,2,3,4,5]. Based on the growing spread of antimicrobial resistance in pathogens causing bovine mastitis and its potential negative consequences for public health, antimicrobial agent use is heavily criticized [6]. A vast majority of all antibiotics used in the dairy industry are applied to control mastitis, mainly at drying-off [6,7,8,9]. Because resistance genes can be found for those antibiotics frequently used for dry cow treatment (DCT), such as β-lactam antibiotics, third-generation cephalosporins, and aminoglycosides [10,11], the European Commission recommends that routine antibiotic treatment of all quarters in all cows at drying-off regardless of their infection status (i.e., blanket dry cow treatment, BDCT) should be avoided. It has been shown, however, that intramammary antibiotic treatment can improve udder health in early lactation [10,11,12,13]. In recent years, various studies have been conducted to compare the efficacy between BDCT and selective dry cow treatment (SDCT) as an alternative concept, where antibiotic treatments are allocated to individual cows or quarters known to have or suspected of having an intramammary infection (IMI). The selection is based on test results of rapid culture systems used before drying-off (culture-guided SDCT, cSDCT) or by using individual cow data, such as somatic cell count (SCC) and history of clinical mastitis (CM) cases, in algorithms (algorithm-guided SDCT, aSDCT) [8,9,14]. Individual studies often encompass a limited number of herds with similar management practices, climatic conditions, and genetic background. This might limit the inference of a treatment effect [15]. The effect of SDCT compared to BDCT on udder health is a hot topic that was recently reviewed by Winder et al. [14] and Kabera et al. [16]. However, both reviews included studies that were conducted as long ago as the 1970s, which might impair the evidence for modern dairy farming, because tremendous changes and improvements have occurred over recent years within the dairy industry regarding animal performance, genetics, nutrition, herd management, and housing [17,18,19]. The structural change seems obvious when considering that there was a 4-fold increase in milk yield between 1944 and 2007, combined with a simultaneous decrease in farm and total cow numbers as well as increase in average herd size [17,20]. Furthermore, progress has been made in mastitis treatment and drying-off management by means of the development of new diagnostic tools such as on-farm culture systems and therapeutic concepts [21]. Pathogens considered responsible for most bovine IMI have also changed over time [5]. Taking this into consideration, the objectives of this paper were (i) to perform a systematic review of the literature over the last 21 yr and (ii) to conduct a meta-analysis to evaluate the efficacy of SDCT vs. BDCT in dairy cows regarding the risk of having IMI after calving, new IMI risk (i.e., infection with a pathogen that was not previously present at drying-off), the cure risk for IMI during the dry period, and the reduction in antibiotic use at drying-off. We hypothesized that SDCT could be an adequate alternative for BDCT regarding udder health that is associated with a simultaneous reduction in antibiotic use.

## 2. Materials and Methods

A review protocol was prepared a priori according to the “Preferred Reporting Items for Systematic Reviews and Meta-Analyses (PRISMA)-P” guidelines [22]. Furthermore, the guidelines used to conduct this systematic review and meta-analysis were based on PRISMA [23] and “A Guide to Conducting Systematic Reviews in Agri-Food Public Health” [24]. The working protocol that was carried out consists of five steps: search strategy, study selection, data extraction, bias assessment, and statistical analysis.

### 2.1. Search Strategy

Database searches were conducted on 10 August 2021 in PubMed (https://pubmed.ncbi.nlm.nih.gov), CAB Direct (https://www.cabdirect.org), and ScienceDirect (http://www.sciencedirect.com), using the search terms “cow” AND “selective” AND “dry cow therapy” OR “dry cow treatment” OR “dry-off” OR “drying-off”. This search strategy was defined based on PICO (Population, Intervention, Comparator, Outcome) terms [25]. Considering the risk of IMI, the risk of new IMI, and antibiotic use as outcomes, the population were dairy cows at drying-off and the intervention was SDCT, while BDCT acted as a comparator. For cure risk during the dry period as an outcome, dairy cows affected by IMI at drying-off were the population of interest, and SDCT and BDCT represented the intervention and comparator, respectively. Furthermore, the reference list of each paper was screened to identify potentially relevant studies that had not been found by database searching. Researchers in the field of bovine udder health were also contacted to obtain additional information regarding potential accepted manuscripts that are not yet published. Duplicate search results were recorded and removed. In cases where data from the same trial were published in ≥2 articles or conference papers, only the most complete manuscript was included. To validate our search strategy, it was ensured that we identified all studies that had been included in the qualitative synthesis of previous systematic reviews dealing with DCT [8,9,14,16].

### 2.2. Study Selection–Inclusion and Exclusion Criteria

Only peer-reviewed manuscripts written in English or German were considered. Furthermore, the search was restricted to studies published after 1999 to ensure that they reflect the circumstances that might be present in modern dairy farms [17,18,19]. Titles and abstracts were initially screened for eligibility. To be selected for data extraction, each manuscript had to meet the following criteria [14,25]:

(1) The study dealt with antimicrobial DCT in dairy cattle and compared SDCT (intervention) vs. BDCT (comparator); case definition of SDCT was individual intramammary antibiotic treatment of cows or quarters that were identified to have IMI or to have a higher risk for IMI, and BDCT was defined as routine intramammary antibiotic treatment of all quarters in all cows at drying-off regardless of their infection status.
(2) The study was a randomized or non-randomized controlled trial with a split herd design.
(3) At least one of the following was a primary outcome of the study: risk of IMI after calving, risk of new IMI after calving, and/or cure risk of IMI during the dry period.
(4) The full text of the manuscript was available.

### 2.3. Data Extraction

Data were collected by the first author (J.W.) using a single electronic form (Excel, Office 2013, Microsoft Deutschland Ltd., Munich, Germany) and validated by two co-authors (J.S. and R.S.). An agreement of two out of these three authors led to an inclusion of the paper. If any discrepancies occurred regarding data extraction, a consensus was obtained among all authors. Regarding study characteristics, the following information was extracted from each article: authors, year of publication, country, study design, milking system, number of herds and animals enrolled, and details of how they meet the inclusion criteria on herd- and cow-level to qualify for enrolment in the study. For animals that received SDCT, data were extracted according to the basis upon which the cows had been selected (i.e., aSDCT vs. cSDCT), and the level of SDCT application (i.e., cow- vs. quarter-level) was recorded. Two studies [26,27] used both aSDCT and cSDCT to select cows for receiving SDCT, resulting in two experimental groups. In this case, we considered each selective treatment arm as a separate experimental group. Consequently, the data of both arms were recorded and analyzed. Data regarding the type and dosage of antimicrobial agents used, as well as the application of internal teat sealants (ITS), were collected for both SDCT and BDCT. Furthermore, IMI case definitions and times of milk sampling at drying-off and after calving were recorded for each study.

### 2.4. Assessment of Risk of Bias in Individual Studies

To determine the risk of bias within the included studies, the “Revised Cochrane risk-of-bias tool for randomized trials” (RoB 2) was used (available at https://sites.google.com/site/riskofbiastool/welcome/rob-2-0-tool/current-version-of-rob-2; accessed on 25 August 2021). Even though the assessment is specific to a single trial outcome, we presented the risk for each of the seven domains for all outcomes of a trial together because all outcomes of a trial revealed the same evaluation result [16]. The assessment was carried out by the first author (J.W.) and validated by two co-authors (J.S. and R.S.). Potential disagreements were resolved by finding a consensus among these authors.

### 2.5. Statistical Analysis

Meta-analysis was performed using MedCalc (version 15.6.1, MedCalc Software, Mariakerke, Belgium). Pairwise meta-analysis was conducted when multiple studies examined the same intervention and comparison [28]. The comparison of interest was between SDCT as the experimental group and BDCT as the control. A random-effects model was used to calculate the relative risk (RR) for cows that received SDCT compared to cows of the BDCT group regarding having an IMI after calving, showing a new IMI after calving, and being cured of an IMI during the dry period. This approach was chosen, as we expected that the true effect sizes would be distributed about some mean among the different studies, because the effect sizes in the studies that actually were conducted are assumed to represent a random sample from a particular distribution of these effect sizes [14,29]. The inconsistency index (i.e., the *I*^2^ statistic) was incorporated to calculate the weighted summary (pooled) RR under the random-effects model [30]. For the effect size of antibiotic use at drying-off (i.e., the percentage of antibiotic treatments within the SDCT groups compared to 100% within the BDCT groups), we calculated the weighted summary proportion under the random-effects model using a Freeman–Tukey transformation [30,31]. The *I*^2^ statistic is used to quantify the degree of heterogeneity, which represents the percentage of observed total variation between studies that is due to real heterogeneity rather than chance. Because negative values of *I*^2^ are transformed to 0, *I*^2^ can range from 0% (indicating no observed heterogeneity) to 100%. If there was substantial heterogeneity (*I*^2^ ≥ 50%), a subgroup meta-analysis was conducted to evaluate potential sources of between-study variation [32]. Forest plots were used to visualize the calculated RR or weighted summary proportion per study and the overall pooled effect of all studies. The forest plots represent the estimated effect size and 95% confidence intervals (CI) in each study along with the study weights [33]. The presence of publication biases was assessed graphically using funnel plots, wherein effect estimates from individual studies were plotted against the estimates’ precision, defined as the inverse of their standard errors. If no bias exists, the plot presents an inverted, symmetrical funnel. In the presence of publication bias, the funnel plot is expected to be skewed [34]. Furthermore, funnel plot asymmetry was measured statistically using Egger’s regression test, which is a test for the Y intercept = 0 from a linear regression of the standardized effect estimate (i.e., the effect estimate divided by its standard error) against the estimate precision [35]. Thus, bias was considered based on graphical asymmetry and *p*-values < 0.100 [34].

## 3. Results

### 3.1. Study Selection

**Figure 1:**
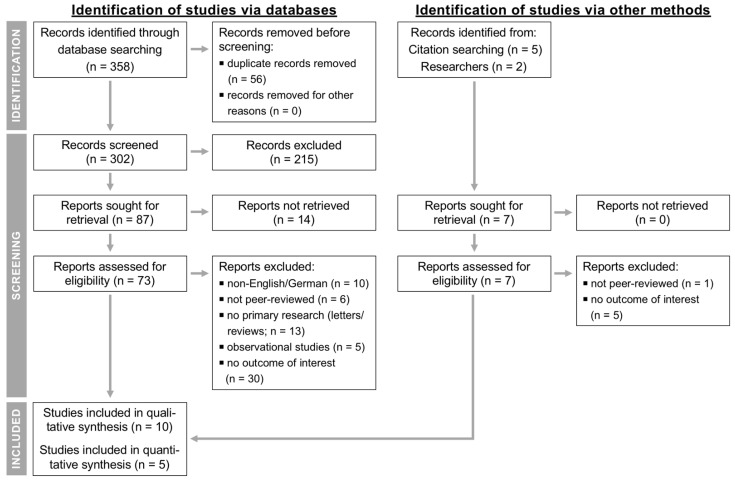
Flow diagram for the systematic review of studies investigating the effect of selective compared to blanket dry cow treatment showing the numbers of studies that were screened, assessed for eligibility, and included in the review and meta-analysis according to the “Preferred Reporting Items for Systematic Reviews and Meta-Analyses” (PRISMA) [23].

Figure 1 shows the PRISMA flow chart [23] providing the numbers of studies that were screened, assessed for eligibility, and included in the review and meta-analysis, with reasons for exclusions at each stage. Out of 309 articles that were initially screened by title and abstract, 80 full texts were reviewed, with 70 studies found to not fulfill the inclusion criteria and the remaining 10 articles being eligible as they investigated the effect of SDCT vs. BDCT on our outcomes of interest. Of these, one paper [36] had to be excluded because some cows eligible for SDCT (low-risk cows) received BDCT due to the study design, and another manuscript [37] did not use BDCT as a comparator and its criteria to select cows for SDCT included aspects of both an algorithm- and culture-based approach, which impairs its comparability to the remaining studies. Furthermore, two papers [38,39] defined SDCT in a different way to us, namely, no treatment of all cows/quarters instead of treatment of all individual cows/quarters having IMI or a higher risk for having IMI, and in another study [40], antibiotic DCT was reserved for cows with an elevated individual cow SCC (ICSCC), which was also in contrast to our BDCT definition. Therefore, data extraction was performed for ≥ 2 outcomes from five studies.

### 3.2. Study Characteristics

A detailed overview of the characteristics of each study is given in Table 1. The trials were performed in three countries: USA (*n* = 2; 40.0%), Canada (*n* = 2; 40.0%), and Germany (*n* = 1; 20.0%). Four studies (80.0%) were found to have a multicenter design, and the remaining trial (20.0%) was designed as a unicenter intervention study. The number of herds varied from 1 to 16, and the number of cows ranged from 56 to 1234. Information regarding the milking system was provided in two out of five (40.0%) papers. The selection criteria for study enrolment on cow- and/or herd-level were reported in all the papers. All the trials were published after 2013.

### 3.3. Outcomes

All the results were given at the quarter-level. The prevalence of IMI after calving was evaluated in three studies, and the prevalence of new IMI after calving and cure risk of IMI during the dry period were recorded in five trials. Diagnosis of IMI postpartum was based on standardized bacteriological methods [46] in all papers. In addition, all studies used bacteriological methods to determine the cure risk over the dry period. Cure over the dry period was additionally assessed cytologically only in the study by tho Seeth et al. [26]. The sampling times and number of milk samples collected varied between studies, ranging from 1 to 18 DIM and from 1 to 2 samples, respectively (Table 2). Even though antibiotic use at drying-off was a key outcome in only three studies, the number of quarter-level antimicrobial treatments within the SDCT groups was given for all trials.

### 3.4. Risk of Bias in Individual Studies

**Figure 2:**
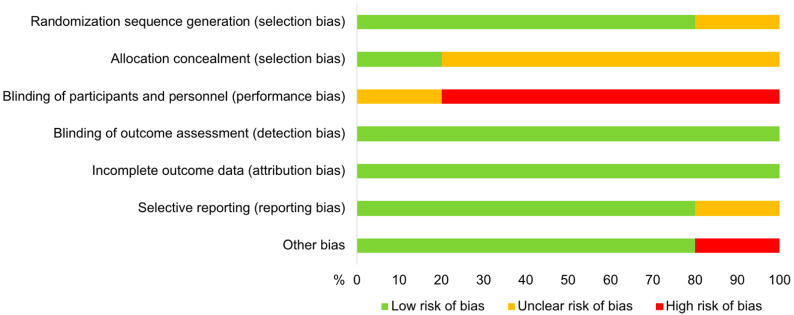
Risk of bias by domain of studies included in the meta-analysis evaluating the effect of selective vs. blanket dry cow treatment on the occurrence of intramammary infections and their cure during the dry period in dairy cows.

The results of the assessed risk of bias within the five studies included in the meta-analysis are shown in Figure 2. All trials revealed at least one potential source of individual study bias. There was a low risk for the occurrence of bias due to blinding of outcome assessment as well as incomplete outcome data in all studies. The method used to generate a random sequence was described for four trials [27,41,42,43]. These four studies also had a low risk for the occurrence of reporting and other bias. The allocation concealment was only described in the study by Kabera et al. [42], whereas the remaining trials were classified as having an unclear risk regarding this domain. Blinding of participants and personnel was not described and therefore the bias risk was determined as “unclear” in the study by Patel et al. [43], and blinding was not performed in the remaining studies, leading to a “high risk” classification.

### 3.5. Pairwise Meta-Analysis

#### 3.5.1. IMI Risk after Calving

**Figure 3:**
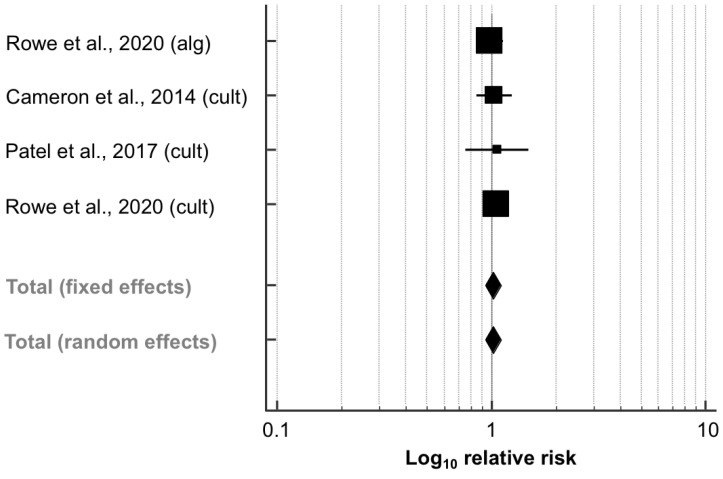
Effect of algorithm-guided (alg) and culture-based (cult) selective dry cow treatment (experimental group) compared to blanket dry cow treatment (control group) on the intramammary infection (IMI) risk after calving. The effect size of each study was summarized into a pooled relative risk (solid squares; marker size relative to study weight) and its 95% confidence interval (CI; whiskers) using a random-effects model. Overall effect size estimates and corresponding 95% CI are represented as the middle of the diamonds and their widths, respectively. Relative risks are plotted on a logarithmic scale, so that effects of the same magnitude but opposite directions are equidistant from 1.

Overall, three studies (4045 quarters) examined the impact of SDCT vs. BDCT on the prevalence of IMI postpartum (Table 3): two studies [41,43] that selected cows for SDCT using an on-farm culture system and one study [27] that used an algorithm- and culture-based approach to select cows for SDCT. As mentioned above, data were extracted from both experimental arms in this study. Figure 3 shows that the RR of having IMI after calving did not differ significantly between SDCT and BDCT based on these three studies (RR, 95% CI: 1.02, 0.94–1.11; *p* = 0.592). No heterogeneity was observed (*I*^2^ = 0.0%; *p* = 0.907). Evidence for the occurrence of publication bias could not be found when evaluating both the funnel plot (Figure S1) and Egger’s regression test (intercept, 95% CI: 0.433, −3.047–3.913; *p* = 0.646).

#### 3.5.2. Risk of New IMI after Calving

**Figure 4:**
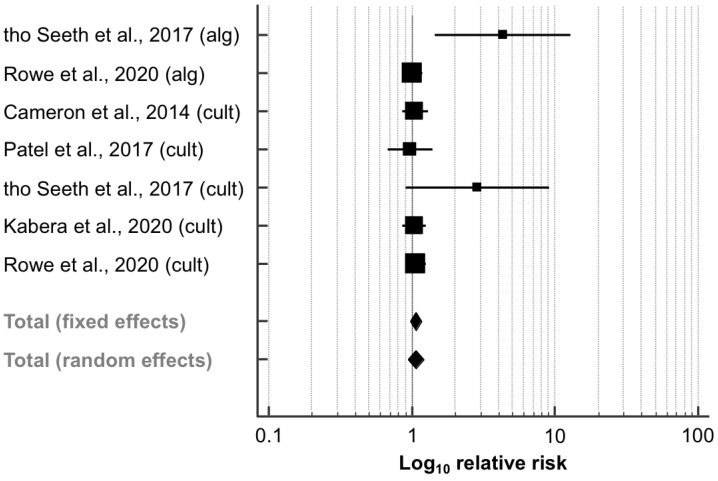
Effect of algorithm-guided (alg) and culture-based (cult) selective dry cow treatment (experimental group) compared to blanket dry cow treatment (control group) on the risk to have a new intramammary infection (IMI) after calving. See Figure 3 for remainder of key.

Data regarding this outcome were extracted from five papers (11,702 quarters; Table 3). Culture-based methods were used in three studies [41,42,43] as selection criteria for the assignment to SDCT. Furthermore, tho Seeth et al. [26] and Rowe et al. [27] designed two experimental groups, each with aSDCT and cSDCT. Data were collected and analyzed for all study arms. In a similar manner to that of IMI risk after calving, the RR of new IMI did not differ significantly between the SDCT and BDCT groups (RR, 95% CI: 1.06, 0.94–1.20; *p* = 0.994; Figure 4). Animals that were allocated to the aSDCT group in the paper by tho Seeth et al. [26] had a significantly higher RR to acquire new IMI (RR, 95% CI: 4.33, 1.46–12.86). Furthermore, there was moderate heterogeneity between the trials (*I*^2^ = 39.5%; *p* = 0.128). The corresponding funnel plot appeared asymmetrical (Figure S2), as also indicated by Egger’s test (intercept, 95% CI: 1.985, 0.401–3.570; *p* = 0.023).

#### 3.5.3. Cure Risk during the Dry Period

**Figure 5:**
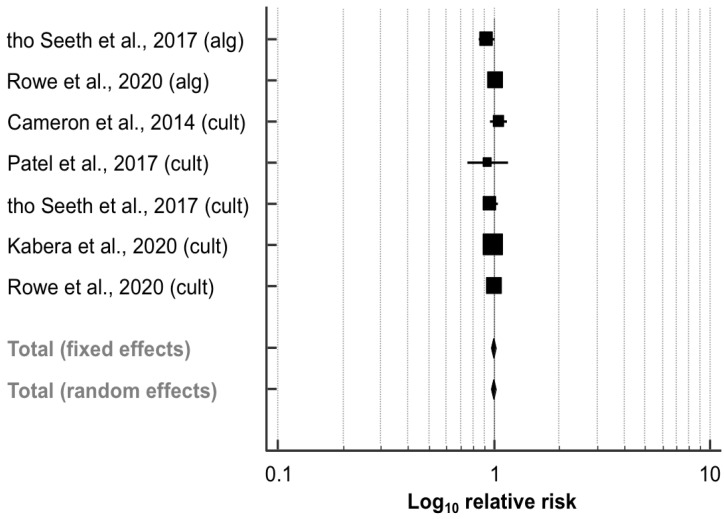
Effect of algorithm-guided (alg) and culture-based (cult) selective dry cow treatment (experimental group) compared to blanket dry cow treatment (control group) on the cure risk during the dry period. See Figure 3 for remainder of key.

Out of the five trials (2785 quarters) comparing the effect of SDCT vs. BDCT on the cure risk during the dry period (Table 3), three studies [41,42,43] performed a culture-based approach to select cows eligible for SDCT, and two papers [26,27] used both algorithm- and culture-based approaches to identify and select cows for receiving SDCT. As described above, the data for each approach were collected and analyzed separately. The meta-analysis revealed that the RR of cure of IMI during the dry period did not differ significantly between SDCT and BDCT (RR, 95% CI: 1.00, 0.97–1.02; *p* = 0.661; Figure 5). No heterogeneity was observed between the seven experimental groups (*I*^2^ = 0.0%; *p* = 0.466). Both the funnel plot (Figure S3) and Egger’s test (intercept, 95% CI: −0.503, −2.859–1.853; *p* = 0.607) revealed no evidence of publication bias.

#### 3.5.4. Antibiotic Use at Drying-Off

**Figure 6:**
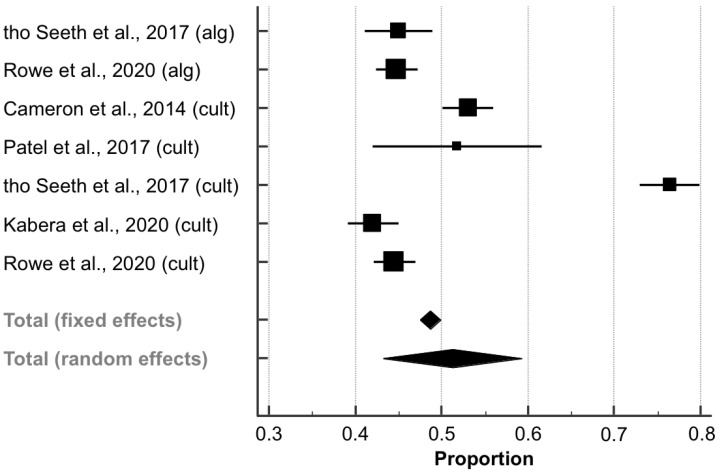
Antibiotic use at drying-off within algorithm-guided (alg) and culture-based (cult) selective dry cow treatment groups. The effect size of each study was summarized into a pooled proportion (solid squares) and its 95% confidence interval (CI; whiskers) using a random-effects model. Overall effect size estimates and corresponding 95% CI are represented as the middle of the diamonds and their widths, respectively.

Data on the proportions of antibiotic use at drying-off within the SDCT groups (compared to 100% within the BDCT groups) were extracted from five papers (6894 quarters; Table 4), as described for the cure risk. The pooled proportion (PP) of antibiotic use within the SDCT groups was 51.22% (95% CI: 43.20–59.22; Figure 6). Furthermore, there was substantial heterogeneity between the studies (*I*^2^ = 97.7%; *p* < 0.001). The corresponding funnel plot showed slightly increased asymmetry (Figure S4), which was not proven to be significant by Egger’s regression test (intercept, 95% CI: 7.431, −13.084–27.946; *p* = 0.395).

### 3.6. Subgroup Meta-Analysis

An additional post-hoc subgroup meta-analysis was performed if meta-analysis revealed an *I*^2^-value of ≥ 50% [32], as this was the case only for antibiotic use, to examine the impact of the method of selecting cows for SDCT (aSDCT vs. cSDCT). because these approaches include more or less sensitive methods for diagnosing IMI [47], the selection method was considered to be a potential source of between-study variation.

The PP of antibiotic use did not differ significantly between the aSDCT and cSDCT subgroups because the CI overlapped (PP, 95% CI; %: 44.85, 42.80–46.91 and 53.84, 42.13–65.35). However, substantial heterogeneity could still be detected within the cSDCT subgroup (*I*^2^ = 98.3%; *p* < 0.001), but not within the aSDCT subgroup (*I*^2^ = 0.0%; *p* = 0.947).

## 4. Discussion

Evidence-based decision making is a hallmark of clinical practice and veterinary public health. Systematic reviews and meta-analytical assessments of randomized controlled field trials provide the highest level of evidence for the efficacy of an intervention [48]. Since the treatment and prevention of bovine mastitis account for a large part of antibiotic use in the dairy industry [6,7], there is growing interest in finding alternative approaches to BDCT. Moreover, SDCT might be a cost-effective practice, as recently shown for US dairy herds by Rowe et al. [49] and Hommels et al. [50]. Compared to previous reviews, the systematic review described in our paper focused on more recent studies which could provide a higher grade of evidence considering the structural changes in dairy farming within the last years. Therefore, results presented in this paper are likely to be valid for current management practices and breeding lines, respectively. The outcomes of this meta-analysis were considered because the risk of new IMI after calving and the cure risk during the dry period reflect the main aims of DCT (i.e., the elimination of existing IMI and prevention of new IMI during the dry period; [8,9]). Furthermore, infection status of the mammary gland during the dry period has been shown to be an important risk factor for developing CM in early lactation and, thus, for increased antibiotic usage [12,13].

### 4.1. Effect of SDCT Compared to BDCT on IMI Risk after Calving

The results showed that the implementation of SDCT did not increase the IMI risk after calving, and there was no heterogeneity between the studies. One explanation for these homogeneous outcomes might be the administration of ITS in each study, which has been shown to reduce the risk of IMI significantly [48,51]. Another reason could be the use of bulk milk SCC as an inclusion criterion on herd-level, as was done by Cameron et al. [41] and Rowe et al. [27], but its use was not reported in the paper by Patel et al. [43]. Consequently, SDCT should only be implemented in farms revealing low bulk milk SCC values, and a general administration of ITS is recommended.

### 4.2. Effect of SDCT Compared to BDCT on New IMI Risk after Calving

Based on our analysis, the use of SDCT also did not increase the risk of acquiring a new IMI during the dry period. The greater RR for cows that received aSDCT to show new IMI after calving in the study by tho Seeth et al. [26] is most likely due to a lack of herd-level inclusion criteria, such as bulk milk SCC, and the cow-level inclusion criteria were less strict than those of the other study that examined aSDCT [27]. This means, again, that there is no reason to fear any detrimental effects of SDCT on new IMI risk postpartum when adhering strictly to these inclusion criteria. Furthermore, there was moderate but not significant heterogeneity between the trials. In this case, a possible cause of heterogeneity could be the length of the dry period: a longer dry period enhances the risk of new IMI due to a lower concentration of the antimicrobial agent present around parturition [8,52]. Unfortunately, this information was not available in many studies, or there was great interindividual variation in dry period length within the study herds. Therefore, further research is needed to examine the impact of dry period length on new IMI risk after calving, preferably using randomized controlled trials. Only then will it be possible to give evidence-based advice to farmers.

### 4.3. Effect of SDCT Compared to BDCT on Cure Risk during the Dry Period

SDCT revealed a similar RR of cure during the dry period compared to BDCT. The use of bacteriological methods to determine the cure risk over the dry period might be one reason for the homogeneity observed between the trials. Furthermore, high apparent spontaneous cure rates in cows showing low ICSCC values at drying-off have been reported [53,54]. However, ICSCC was used as a selection criterion for study enrolment or for receiving SDCT in only three out of five trials. Moreover, Vasquez et al. [36] found that there was also a high self-cure rate during the dry period for IMI caused by non-aureus staphylococci, accounting for a major proportion of IMI during the dry period [27]. In this context, it remains unclear whether or to what extent quarters infected with minor pathogens benefit from antimicrobial treatment. Further studies are, therefore, necessary to evaluate the effects of targeted, pathogen-specific antimicrobial DCT on udder health. This aspect seems to be particularly important in terms of both economic reasons and a responsible use of antimicrobial agents.

### 4.4. Antibiotic Use at Drying-Off within the SDCT Groups

Considering all five studies, the PP of antibiotic use within the SDCT groups was 51.2%, which means that the implementation of SDCT can reduce antimicrobial treatments by half compared to BDCT, where the proportion of antibiotic use, by definition, is 100%. However, antibiotic use was significantly higher within the cSDCT group in the study by tho Seeth et al. [26]. Furthermore, substantial heterogeneity was seen within both pairwise and subgroup analysis, suggesting that the true effect size (i.e., the proportion of antibiotic use) differs from the calculated point estimates. Interestingly, no heterogeneity was observed within the aSDCT subgroup. This subgroup, however, included only two studies. The method used to determine infected cows (aSDCT vs. cSDCT) may explain some of the heterogeneity detected between these studies, alongside other unmeasured risk factors. Several studies have shown that algorithms are not sensitive predictors of IMI caused by minor pathogens (e.g., *Corynebacterium* spp., coagulase-negative or non-aureus staphylococci) because these infections may fail to increase the SCC above its commonly used thresholds [55]. This fact might also explain why there were more antibiotic treatments (due to more IMI detected) within the cSDCT group in the paper by tho Seeth et al. [26]. Moreover, the mean bulk milk SCC of the herds included in this study was 280,000 SC/mL (range: 227,000–334,000 SC/mL), and there were less stringent inclusion criteria on the cow-level, as mentioned above, suggesting that there might have been a higher proportion of cows with IMI.

### 4.5. Methodological Strengths and Limitations

All the included studies were randomized controlled trials that, along with systematic reviews, provide the highest level of evidence [24,56]. Our systematic review was restricted to this type of study by design. Nevertheless, observational studies and challenge trials can also be used for evaluating on-farm interventions [24]. This restriction led to a small number of studies being used for the meta-analysis, constituting a clear limitation. However, using more studies for quantitative data synthesis by the application of less stringent inclusion criteria, as was done in previous reviews, increases the risk for comparing apples and oranges, or in other words, heterogeneity between the trials [57]. Even if we strongly implemented strict inclusion criteria, different sampling times and numbers of collected milk samples between the studies included in this meta-analysis might impair the generalizability of our results. Besides, predominant circulation of different mastitis pathogens (i.e., contagious pathogens that are known to spread among cows vs. environmental pathogens) between the herds might be a source of heterogeneity [5]. In contrast, the pp IMI status was determined using standard bacteriological methods in all included studies, and therefore the risk of between-study variation due to different diagnostic methods seems to be low. Another reason why this paper may not reflect the entire peer-reviewed literature is linguistic limitations, a common feature of most reviews [58]. However, considering the exclusion percentages regarding study design (1.4%) and language (2.8%), the probability of missing an important article is quite low. Furthermore, because our review focused on trials conducted over the last 21 yr, it reflects more recent housing and management practices that are more relevant for modern dairy farming [17,18,19], and can therefore, in our opinion, provide an increased grade of evidence for current conditions. This approach was also adopted as part of an attempt to improve homogeneity, as can be seen regarding the application of ITS, which were not commonly used in studies conducted before the year 2000 [51]. Possible further sources of between-study heterogeneity with respect to all outcomes include variations in the IMI definition and sampling time. Because of the heterogeneous nature of the included studies, we decided to calculate the pooled RR or PP by assuming a distribution of the true RR or proportion, in which weighting is applied differently (i.e., random-effects model [29]). Another limitation of this meta-analysis is that the definitions of IMI and new IMI were slightly different between the trials. Future studies are needed to evaluate the effect of SDCT compared to BDCT on udder health and animal performance (e.g., ICSCC, CM incidence, milk yield) in subsequent lactation. Those studies should preferably be designed as randomized controlled trials and follow the “Reporting Guidelines for Randomized Controlled Trials in Livestock and Food Safety” (REFLECT) to ensure more consistency between the trials, enabling more precise overall effect size estimates [59,60]. Finally, all studies used rapid on-farm culture systems to identify cows for receiving cSDCT, but there is a lack of studies comparing laboratory bacteriological methods with the use of rapid culture systems and algorithm-based selection methods. Therefore, it cannot be excluded that the application of classical bacteriology as a selection criterion could show better effects on these outcomes than algorithms or rapid culture systems, even if laboratory bacteriological methods are rarely used at drying-off due to the increased logistical effort required. Besides this, selection based on results of the California mastitis test as a semiquantitative screening test for estimating current ICSCC data compared to ICSCC determined during the last dairy herd improvement tests should be investigated in further studies.

## 5. Conclusions

This meta-analysis provides evidence that SDCT seems to be an adequate alternative to BDCT by showing similar effects on IMI risk after calving, risk of new IMI after calving, and cure risk during the dry period. Furthermore, the implementation of SDCT can help to decelerate the spread of resistance genes by reducing antibiotic usage. Considering all outcomes of udder health under investigation, the method used to select cows eligible for SDCT (algorithm- vs. culture-guided) did not influence its efficacy. However, more research is needed to explore further herd- and cow-level risk factors explaining between-study variation and to investigate the use of classical bacteriology to select cows for cSDCT compared to algorithm-based approaches or rapid on-farm culture systems. The concurrent application of ITS at drying-off should be a mainstay of good veterinary practice.

## Figures and Tables

**Table 1:** Characteristics of manuscripts included in the meta-analysis evaluating the effect of selective vs. blanket dry cow treatment on the occurrence of intramammary infections and their cure during the dry period in dairy cows.

			Study Inclusion Criteria				ITS	
Study	Country ^1^	No. Herds/ Animals	Herd-Level	Cow-Level	Selection Criteria for SDCT	Antimicrobial Agent ^2^	BDCT	SDCT
Cameron et al. [41]	CA	16/729	mean BMSCC < 250,000 SC/mL over the last 12 mo	≥3 functional quarters, DP of 30–90 d, monthly ICSCC < 200,000 SC/mL on the last 3 DHIT, no CM or antibiotic treatment within last 14 d, CMT score < 2 in all quarters at drying-off	cSDCT (3M Petrifilm: Growth of ≥5 colonies after 24 h of incubation vs. no growth; cow-level)	500 mg of ceftiofur	+	+
Kabera et al. [42]	CA	9/568	mean BMSCC < 250,000 SC/mL over the last 12 mo, DHIT recording	≥3 functional quarters, no CM/antibiotic treatment within last 14 d, DP of 35–75 d	cSDCT (3M Petrifilm: Growth of ≥5 colonies after 24 h of incubation vs. no growth; quarter-level)	200,000 IU of procaine benzylpenicillin and 400 mg of novobiocin	+/−	+/−
Patel et al. [43]	US	1/56	not reported	4 functional quarters, clinical healthy, no antibiotic or anti-inflammatory treatment within last 14 d, DP of 30–90 d	cSDCT (Minnesota Easy 4Cast plate: Growth of ≥100 cfu/mL of milk after 36 h of incubation vs. no growth; quarter-level)	500 mg of ceftiofur	+	+
Rowe et al. [27]	US	7/1234	DCT in 15 cows/wk, mean BMSCC < 250,000 SC/mL over the last 12 mo, monthly DHIT recording, recording of CM and culling	4 functional quarters, DP of 30–90 d, no antibiotic or anti-inflammatory treatment within last 14 d, no CM, locomotion score < 4 ^3^, BCS > 1 ^4^	cSDCT (Minnesota Easy 4Cast plate: Growth after 30–40 h of incubation vs. no growth; quarter-level) and aSDCT (≥2 cases of CM or any DHIT showing ICSCC > 200,000 SC/mL during preceding lactation; cow-level)	500 mg of ceftiofur	+	+
tho Seeth et al. [26]	GE	4/482	not reported	no CM at drying-off	cSDCT (3M Petrifilm: Growth of ≥5 colonies after 24 h of incubation vs. no growth; cow-level) and aSDCT (ICSCC ≥ 200,000 SC/mL during the last DHIT or CM cases during the previous lactation; cow-level)	not specified	+	+

^1^ CA = Canada; GE = Germany; US = United States. ^2^ Antimicrobial agents used for SDCT and BDCT, respectively. ^3^ According to Sprecher et al. [44]. ^4^ According to Edmonson et al. [45]. aSDCT = algorithm-guided SDCT; BDCT = blanket dry cow treatment; BMSCC = bulk milk somatic cell count; CM = clinical mastitis; CMT = California mastitis test; cSDCT = culture-guided SDCT; DCT = dry cow treatment; DHIT = dairy herd improvement test; DP = dry period; ICSCC = individual cow somatic cell count; ITS = internal teat sealant; SDCT = selective dry cow treatment.

**Table 2:** Definitions of intramammary infections (IMI) and new IMI including sampling times and number of milk samples collected for each study included in the meta-analysis.

Study	Case Definitions and Times of Sampling	
	IMI	New IMI
Cameron et al. [41]	growth of a pathogen on culture at 3–4 DIM and/or at 5–18 DIM	pathogen was present in the pp samples that was not present in the drying-off sample
Kabera et al. [42]	not determined	growth of a pathogen on culture at 3–4 DIM and/or at 5–18 DIM that was not present in the drying-off sample
Patel et al. [43]	detection of ≥100 cfu/mL of milk for any organism (except *Bacillus* spp. and CNS) in a sample taken at 1–7 DIM	growth of 1–2 pathogens in the pp sample that were not present in the drying-off sample
Rowe et al. [27]	growth of a pathogen on culture using 2 samples taken within the first 13 DIM	growth of a pathogen in the pp samples that was not present in the drying-off sample
tho Seeth et al. [26]	not determined	growth of a pathogen on culture using samples collected at 3–10 DIM and at 11–18 DIM that was not present in the drying-off sample

CNS = coagulase-negative staphylococci; DIM = days in milk; pp = postpartum.

**Table 3:** Effect of selective compared to blanket dry cow treatment on intramammary infection (IMI) risk after calving, new IMI risk after calving, and cure risk during the dry period.

Outcome	Study	SDCT ^1^	BDCT ^1^	Relative Risk ^2^	95% CI	*p*-Value
IMI risk	Rowe et al. [27] ^3^	303/1355	317/1392	0.98	0.86–1.13	
	Cameron et al. [41] ^4^	179/1130	177/1157	1.04	0.86–1.25	
	Patel et al. [43] ^4^	43/102	36/91	1.07	0.76–1.50	
	Rowe et al. [27] ^4^	341/1426	317/1392	1.05	0.92–1.20	
	*I*^2^ = 0.0% ^5^ (*p* = 0.907)					
	Total (fixed effects)	866/4013	847/4032	1.02	0.94–1.11	0.600
	Total (random effects)	866/4013	847/4032	1.02	0.94–1.11	0.592
New IMI risk	tho Seeth et al. [26] ^3^	16/566	4/612	4.33	1.46–12.86	
	Rowe et al. [27] ^3^	246/1241	246/1255	1.01	0.86–1.19	
	Cameron et al. [41] ^4^	164/1130	160/1157	1.05	0.86–1.28	
	Patel et al. [43] ^4^	39/97	34/82	0.97	0.68–1.38	
	tho Seeth et al. [26] ^4^	10/534	4/612	2.87	0.90–9.08	
	Kabera et al. [42] ^4^	163/899	169/964	1.03	0.85–1.26	
	Rowe et al. [27] ^4^	272/1298	246/1255	1.07	0.92–1.25	
	*I*^2^ = 39.5% ^5^ (*p* = 0.128)					
	Total (fixed effects)	910/5765	863/5937	1.06	0.98–1.15	0.169
	Total (random effects)	910/5765	863/5937	1.06	0.94–1.20	0.320
Cure risk	tho Seeth et al. [26] ^3^	119/140	161/176	0.93	0.86–1.01	
	Rowe et al. [27] ^3^	267/303	263/303	1.02	0.96–1.08	
	Cameron et al. [41] ^4^	152/171	121/143	1.05	0.96–1.15	
	Patel et al. [43] ^4^	28/34	22/25	0.94	0.76–1.16	
	tho Seeth et al. [26] ^4^	105/119	161/176	0.97	0.89–1.04	
	Kabera et al. [42] ^4^	240/251	300/312	0.99	0.96–1.03	
	Rowe et al. [27] ^4^	288/329	263/303	1.01	0.95–1.07	
	*I*^2^ = 0.0% ^5^ (*p* = 0.466)					
	Total (fixed effects)	1199/1347	1291/1438	1.00	0.97–1.02	0.766
	Total (random effects)	1199/1347	1291/1438	1.00	0.97–1.02	0.661

^1^ No. of quarters (events/total). ^2^ Ratio of risk of IMI, new IMI, and cure during the dry period in cows that received SDCT to risk of IMI, new IMI, and cure during the dry period in cows that received BDCT. ^3^ Algorithm-guided SDCT. ^4^ Culture-guided SDCT. ^5^ Proportion of total variation of effect size estimates that is due to heterogeneity rather than chance. BDCT = blanket dry cow treatment; CI = confidence interval; SDCT = selective dry cow treatment.

**Table 4:** Proportion of antibiotic use at drying-off (AB+) within the selective dry cow treatment groups including 6894 quarters.

Study	No. Quarters	Quarters AB+ (%)	95% CI
tho Seeth et al. [26] ^1^	634	44.95	41.03–48.92
Rowe et al. [27] ^1^	1616	44.8	42.36–47.27
Cameron et al. [41] ^2^	1130	53.1	50.14–56.04
Patel et al. [43] ^2^	108	51.85	42.03–61.57
tho Seeth et al. [26] ^2^	604	76.49	72.90–79.82
Kabera et al. [42] ^2^	1114	42.01	39.09–44.97
Rowe et al. [27] ^2^	1688	44.55	42.16–46.96
*I*^2^ = 97.7% ^3^ (*p* < 0.001)			
Total (fixed effects)	6894	48.67	47.48–49.85
Total (random effects)	6894	51.22	43.20–59.22

^1^ Algorithm-guided selective dry cow treatment. ^2^ Culture-guided selective dry cow treatment. ^3^ Proportion of total variation of effect size estimates that is due to heterogeneity rather than chance.

## Data Availability

None of the data were deposited in an official repository.

## Supplementary Materials

The following are available online at https://www.mdpi.com/article/10.3390/ani11123403/s1, Figure S1: Funnel plot of studies involved in the estimation of the relative risk of IMI after calving. Smaller standard errors result from trials with a larger number of cows enrolled in the trial (i.e., they are inversely proportional to the number of animals). Outer diagonal lines indicate the triangular area within which 95% of studies are expected to be located in the absence of heterogeneity and bias, respectively. The presence of publication bias will cause an asymmetrical appearance of the plot. In this case, the (inverted) funnel shows no asymmetry, and Egger’s test did not indicate publication bias (*p* > 0.100), Figure S2: Funnel plot of studies involved in the estimation of the relative risk of developing an IMI after calving. The funnel appears symmetrically, indicating no existence of publication bias. Publication bias could also not be found using Egger’s test (*p* < 0.100). See Figure S1 for remainder of key, Figure S3: Funnel plot of studies involved in the estimation of the relative risk to cure from an intramammary infection during the dry period. The funnel is symmetrical, showing no evidence for the existence of publication bias. Similarly, Egger’s test did not indicate publication bias (*p* > 0.100). See Figure S1 for remainder of key, Figure S4: Funnel plot of studies involved in the estimation of the proportion of antibiotic use at drying-off within the selective dry cow treatment groups. The funnel shows moderate asymmetry, but Egger’s test did not indicate publication bias (*p* > 0.100). See Figure S1 for remainder of key.
