# Supplementary material for: Effects of Selective Dry Cow Treatment on Intramammary Infection Risk after Calving, Cure Risk during the Dry Period, and Antibiotic Use at Drying-Off: A Systematic Review and Meta-Analysis of Current Literature (2000–2021)
Source: Animals (Basel). 2021 Nov 29;11(12):3403. doi: 10.3390/ani11123403 (PMC8698164; doi:10.3390/ani11123403)
Supplement: Supplementary file 1 [file animals-11-03403-s001.zip › animals-1456309-supplementary.pdf]

Supplementary Material

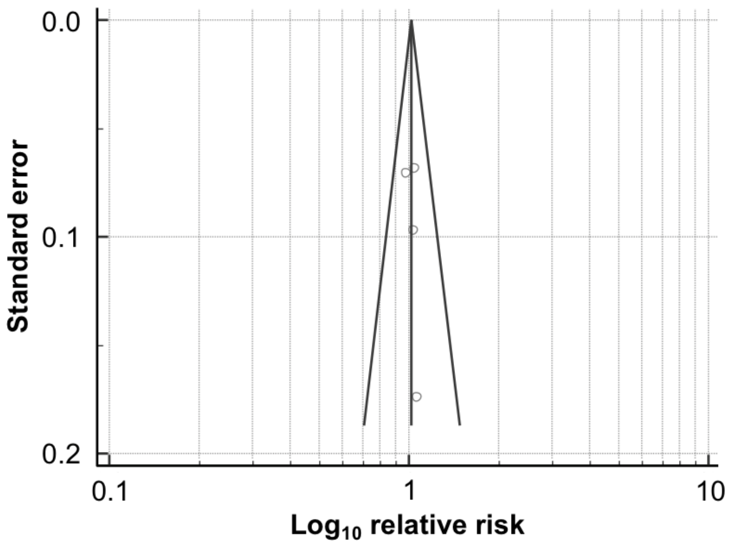

Figure S1: Funnel plot of studies involved in the estimation of the relative risk of IMI after calving. Smaller standard errors result from trials with a larger number of cows enrolled in the trial (i.e., they are inversely proportional to the number of animals). Outer diagonal lines indicate the triangular area within which 95% of studies are expected to be located in the absence of heterogeneity and bias, respectively. The presence of publication bias will cause an asymmetrical appearance of the plot. In this case, the (inverted) funnel shows no asymmetry and Egger's test did not indicate publication bias (*P* > 0.100).

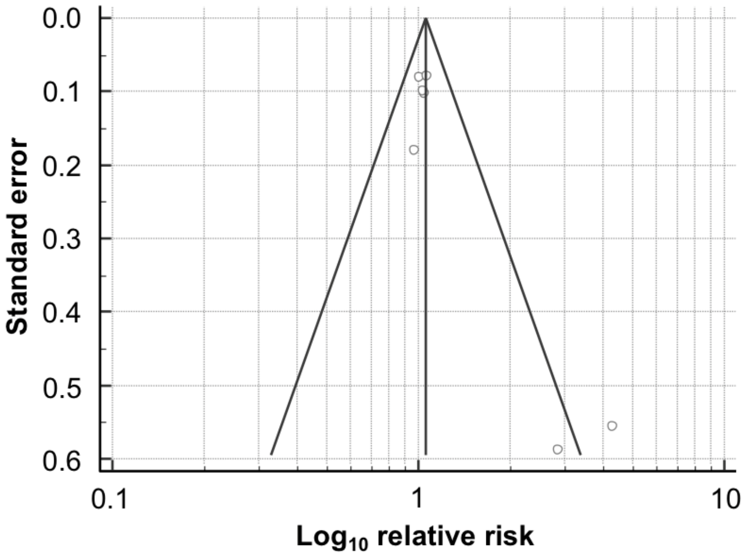

Figure S2: Funnel plot of studies involved in the estimation of the relative risk of developing an IMI after calving. The funnel appears symmetrically, indicating no existence of publication bias. Publication bias could also not be found using Egger's test (*P* < 0.100). See Figure S1 for remainder of key.

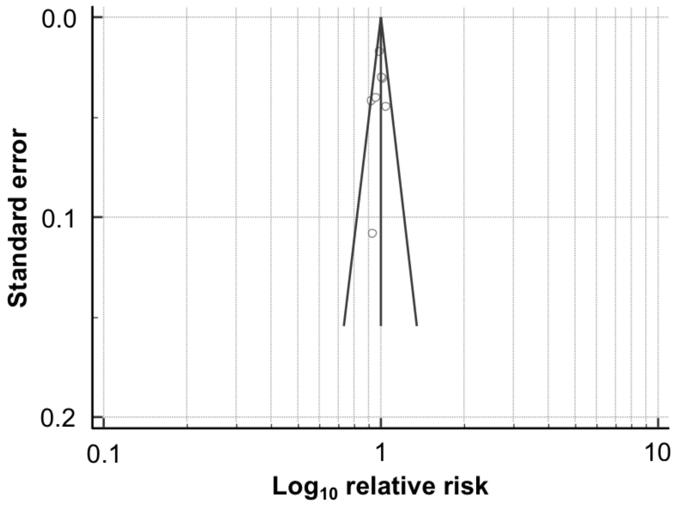

Figure S3: Funnel plot of studies involved in the estimation of the relative risk to cure from an intramammary infection during the dry period. The funnel is symmetrical showing no evidence for the existence of publication bias. Similarly, Egger's test did not indicate publication bias (*P* > 0.100). See Figure S1 for remainder of key.

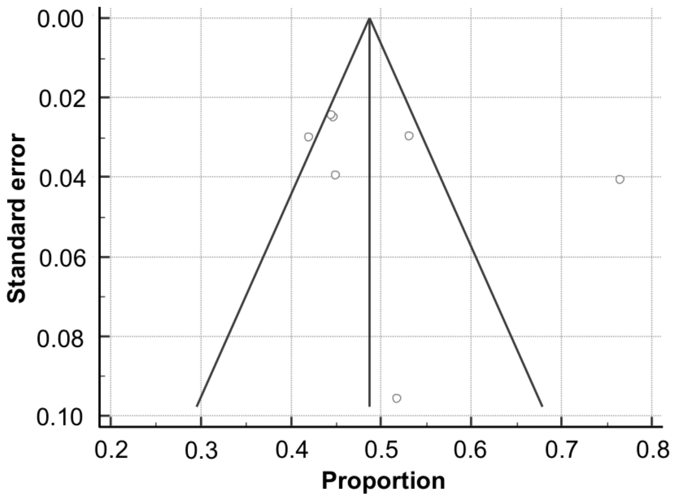

Figure S4: Funnel plot of studies involved in the estimation of the proportion of antibiotic use at drying-off within the selective dry cow treatment groups. The funnel shows moderate asymmetry but Egger's test did not indicate publication bias (*P* > 0.100). See Figure S1 for remainder of key.
